# Mucinous eccrine carcinoma of the eyelid: A case report study

**DOI:** 10.22088/cjim.11.3.337

**Published:** 2020-05

**Authors:** Etrat Javadirad, Javad Azimivaghar, Saba montazer

**Affiliations:** 1 Department of Pathology, School of Medicene, Kermanshah University of Medical Sciences, Kermanshah, Iran; 2 Department of Cardiology, School of Medicene, Kermanshah University of Medical Sciences, Kermanshah, Iran

**Keywords:** Eccrine, Mucinous, carcinoma, Eye

## Abstract

**Background:**

Primary mucinous eccrine carcinoma (PMEC) is a quite rare malignant tumor that typically involves the head and neck region in approximately 75% of patients and the periorbital area is the most common area.

**Case Presentation:**

A 57-year-old man was seen with a painless red mass involving the left lower eyelid for the past 7 months. Examination revealed a small mass measuring 0.6 cm with shiny red smooth appearance of the skin. H&E stained examination revealed a tumor mass that composed of polygonal cells in nests, lobules and islands separated by large amount of mucin. The neoplastic cells showed eosinophilic cytoplasm and round nuclei with mild pleomorphism. There was no necrosis, no atypical mitosis, no lymphovascular and perineural invasion. Rare mitotic figures were found. Tumoral nests present on all surgical margins. Primary MEC is a slow-growing neoplasm that may recur after incomplete surgical excision. This tumor is often clinically mistaken for other cutaneous tumors due to its variable appearance. Recurrent tumor tends to be locally invasive with a rare metastatic rate of 9.6%.

**Conclusion:**

As a result of the recurrence risk, patients should be followed up regularly. Thus, our patient was recommended to have a regular follow-up every six months.

Primary mucinous eccrine carcinoma is an uncommon neoplasm of sweat gland origin (1, 2). It is a low-grade malignant tumor that was first described by Mendoza *et al*. as mucinous carcinoma of skin (1, 2). This tumor usually affects old patients with slightly male preponderance and mean age 50 years (range 30–78 y) at presentation time (3). It may be locally invasive and recur after incomplete surgical excision, however its metastasis is quite rare (3). This neoplasm can arise at the head and neck region, scalp, eyelids, trunk and axilla (1, 3). The clinical presentation in most cases is a slow-growing lesion that manifests over months to years. Most lesions appear as a solitary nodule that measure from 2 milimeters to few centimeters in diameter.Hematoxylin and eosin stained sections histologic study of primary mucinous eccrine carcinoma usually mimics metastatic carcinoma of gastrointestinal tract, ovary, breast or lung origin and results in diagnostic confusion (2, 4). In this study, we report a very rare case of primary mucinous eccrine carcinoma of eyelid in a 57-year-old male. 

## Case Presentation

A painless nodule it was a painless nodule locked in the periorbital region. On local examination, a firm, non-tender, mobile nodule measuring 9x6 mm without localized or generalized lymphadenopathy was found. He had a controlled hypertension but no history of weight loss or smoking. All routine biochemical, hematological tests and chest x-ray were within normal limit.

**Figure1:**
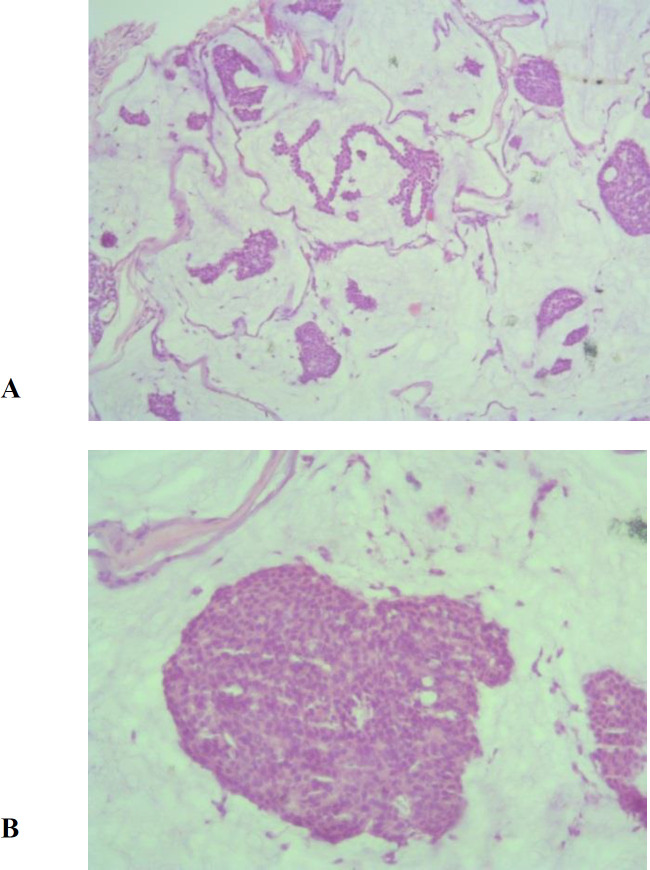
a- .Histopathological section of skin lesion of the patient showing pools of mucin, divided into compartments by collagenous septae and islands of tumor cells floating within it (H & E, x100) b -Islands of tumor cells showing mild cytological atypia and focal duct formation (H & E, x400)

Gross examination of the specimen showed a partially skin covered solid irregular gelatinous tissue bit measured 8x5 mm. Histopathology revealed pools of mucin arranged in lobules, separated by collagenous septae. Epithelial tumoral nests were floating in the mucin pools, arranged in small cluster islands and tubular patterns (fig.1 a,b). Individual tumor cells showed scant eosinophilic, polygonal cytoplasm with hyperchromatic round nuclei and mild pleomorphism. There were no tumoral necrosis, perineural and lymphovascular invasion.Very scanty mitotic figures were noted .Surgical margins were involved in tumor. Immunohistochemically, the tumor tissue was positive for estrogen receptor (ER), epithelial membrane antigen (EMA) and cytokeratin 7 (CK-7), diffusely. Final diagnosis of mucinous eccrine carcinoma was made and the patient was referred to an ophthalmology center for further workup and wide local tumor excision.

## Discussion

Primary mucinous eccrine carcinoma (PMEC) is a very rare malignant cutaneous adnexal slow-growing tumor which is controversial regarding whether it arises in a eccrine sweat gland or apocrine gland (1, 3). This tumor commonly arises in the head or neck region, that the eyelid is the most commonly involved site (41%) which is usually present as a unilateral mass, however, few bilateral cases have been reported. Other involved sites are scalp (17%), trunk, face (14%) and axillae. Males are more affected than female (1, 2, 5). PMECs occur frequently in elderly (average age 50y, range 30–78 y) (2, 3). These cutaneous mucinous carcinomas usually present as solitary, painless, nodular lesions ranging from 2mm to 12cm in size. The tumoral lesion can have an ulcerated, smooth or crusted surface (1, 3, 4, 5).

The first case was reported by Mendoza *et al*. in 1971(1). These tumors present with a variety of clinical appearance, thus several clinical differential diagnoses are considered including benign and malignant cutaneous lesions such as chalazion, pyogenic granuloma, epidermoid cyst, lipoma, hemangioma, myxoma, keratoacanthoma, sebaceous cyst, sebaceous carcinoma, BCC, SCC, adenoid cystic carcinoma and etc (5, 6, 7). However, the most important microscopic differential diagnosis- metastatic mucinous carcinoma from the other site - has to be ruled out (8). The tumor microscopic clue to an intestinal origin is an evidence of necrosis and the presence of epithelial cells showing goblet cell differentiation (3, 5, 8). Our present case did not reveal these findings which helped in ruling out a primary source in the gastrointestinal tract.

The local recurrence rate and metastatic rate of PMEC are 29.4% and 9.6%, respectively (9, 10). Majority of metastases are regional lymph nodes. Skeletal metastasis has been reported in about 7% cases (9, 11). According to literature in three cases, mortality occurs due to tumor metastasis (9, 10, 12). No metastasis was found in the present case. The recommended treatment modality in PMEC is awide local excision with at least 1 cm margin (12). This tumor is chemo- and radio-resistant (9, 11). However, prognosis is very good in comparison to secondary mucinous carcinomas. As a result of the recurrence risk, patients should be followed up regularly, and also it is possible that tumors without in- situ elements might represent a metastasis (8, 9, 12). Thus, our patient was recommended to have a regular follow-up every six months. In conclusion according to this study, pathologists are required to focus on the rarity and location of this malignant neoplasm and a suspicion to make final diagnosis of this uncommon entity which actually mimics metastatic mucinous carcinoma. Primary MEC is a slow-growing tumor that may recur after traditional surgical excision and recurrent eyelid MEC tends to be locally destructive. Thus, we recommend complete surgical excision of the eyelid PMEC with histologic monitoring of the resected surgical margins. Proper patient follow-up is essential for treatment and cooperation between clinicians and pathologists, for best therapeutic results, is necessary.
